# The Effects of Wearing a Removable-Partial-Denture on the Bite Forces: A Cross-Sectional Study

**DOI:** 10.3390/ijerph182111401

**Published:** 2021-10-29

**Authors:** Iole Vozza, Licia Manzon, Pier Carmine Passarelli, Nicola Pranno, Ottavia Poli, Cristina Grippaudo

**Affiliations:** 1 Department of Oral and Maxillofacial Sciences, Sapienza University of Rome, 00168 Rome, Italy; licia.manzon@uniroma1.it (L.M.); nicola.pranno@uniroma1.it (N.P.); ottavia.poli@uniroma1.it (O.P.); 2 Department of Head and Neck, Division of Oral Surgery and Implantology, Catholic University of the Sacred Heart, Fondazione Policlinico Gemelli IRCCS, 00168 Rome, Italy; piercarmine.passarelli@unicatt.it (P.C.P.); cristina.grippaudo@policlinicogemelli.it (C.G.)

**Keywords:** bite force, removable prostheses, partial denture, chewing strength, body mass index

## Abstract

Background: Removable partial dentures are a frequently used prosthetic treatment in the elderly population, but different types or RPDs might guarantee different chewing capabilities. In many studies, the relationship between chewing and aging has been reported and it has been shown that efficient chewing can improve the overall quality of life. Objectives: In the present study, the relationship between maximum bite force (MBF) and RPDs was studied. A relationship between the body mass index (BMI) and the type of prosthesis was also analyzed. Methods: 240 elderly patients, 120 males and 120 females, with bilateral posterior edentulism (class 1 of Kennedy classification) who had been wearing an RPD for at least a year, were recruited. Patients were divided into two groups: Group 1: male (*n* = 60) and female (*n* = 60) patients with bilateral edentulous areas located posterior to the remaining natural teeth and natural teeth in the opposite dental arch. Group 2: male (*n* = 60) and female (*n* = 60) patients with maxillary and mandibular bilateral edentulous areas located posterior to the remaining natural teeth. Their Body Mass Index (BMI) and Maximum bite force (MBF) were measured and compared according to the material and design of their RPD. Results: In both Groups, patients wearing cobalt-chrome alloy RPDs (Co-Cr-RPD) (Group 1: 20.25 ± 6.7 MBF, *p* < 0.001; Group 2: 16.0 ± 5.7 MBF, *p* < 0.001) had an increased MBF when compared to polymethylmethacrylate RPD (PMMA-RPD) (Group 1: 12.9 ± 3.36 MBF; Group 2: 10.4 + 2.8 MBF), and Valplast RPD (V-RPD) (Group 1: 14.3 ± 4.7 MBF; Group 2: 11.3 ± 3.4 MBF) users. There were no significant differences in bite force between patients wearing PMMA-RPD and V- RPD in both Groups. Patients in Group 2 showed a lower MBF than those in Group 1 (Group 1: 16.05 ± 6.13 MBF; Group 2: 12.6 ± 4.84 MBF; *p* < 0.001). Conclusions: A reduction in chewing force can lead to choosing softer foods for nutrition, which can lead to an increase in BMI. Our results show that only CoCr-RPD wearers were able to chew consistent food, whereas PMMA- RPD and V-RPD, due to the properties of the materials, their instability, and the possibility of causing pain during mastication, determined a limitation in the choice of food for many of the participants.

## 1. Introduction

Removable partial dentures (RPDs) are often used for treating partial edentulism in elderly patients, who cannot afford more complex procedures or won’t accept an implant-supported rehabilitation. These rehabilitations provide these patients with the needed occlusal contacts, which are needed for maintaining an appropriate chewing efficiency, improving the patients’ quality of life, and keeping a healthy eating plan [1].

Conventional RPDs are either made of polymethyl methacrylate (PMMA-RPD) and incorporate stainless steel clasps or are made of a cast metal alloy (CoCr-RPD) [2].

Injection-molded thermoplastic materials (such as Valplast) are an alternative to PMMA. They have a lower elastic module when compared to PMMA [3], their color may deteriorate in time, they can easily deform and the bond of the artificial teeth to the resin is not as strong as PMMA [4]. Still, these materials are very popular as clasps can be made of the same material, they are more esthetic, and they allow for slimmer prostheses that are much more flexible [5]. All these properties make RPDs made from Valplast (V-RPD) more comfortable to wear and remove [6].

Traditional RPDs are frequently deemed as unsatisfactory mainly because of their visible and unsightly clasps and rests and their encumbrance, the type of prosthesis that can influence the effectiveness of chewing, and studies have shown that chewing performance, bite forces and masticatory abilities are lower in patients wearing a removable prosthesis, compared to people with natural teeth or fixed prosthesis [7]. The maximum bite force (MBF) is the most reliable index of occlusal force, and it is used to assess the functional state of the masticatory system [8,9].

The association between nutritional status (BMI) and dentures has been validated, but there are few studies on the effect of partial dentures on bite force and nutritional status in the elderly, despite the high and growing number of elderly patients wearing RPD [10].

This study investigated the BMI and the MBF exerted by the elderly (≥65 years old) using a CoCr-RPD and/or PMMA-RPD and/or V-RPD. Being that these different kinds of RPD strongly affect the MBF and occlusal forces; the subject’s food choice, and subsequently their BMI, could be affected by this and the relation was studied.

## 2. Materials and Methods

The present study protocol was accepted by the ethical committee of Sapienza University of Rome, with protocol code 0653/2020. Informed consent was obtained from all subjects involved in the study.

Subjects were selected from the patients of the Department of Geriatric Dentistry at Umberto I Hospital in Rome, according to our inclusion criteria.

It was not possible to randomize the sample because priority was given to clinical, aesthetic and economic criteria for the choice of prosthesis on a patient-by-patient basis.

In order to observe the RPD effects on MBF, patients that had only one RPD (in the maxillary or in the mandibular arch) and patients that had two RPDs (one in the maxillary and one in the mandibular arch) were enrolled.

Defining the effect of different designs of removable prostheses on MBF as the main outcome, the sample size was calculated using an expected effect size of 0.4, with a significance threshold set at 0.05 and a test power of 0.95. Therefore, the minimum sample size required was 102 subjects per Group (based on the mean values reported by the first 15 patients through G*Power (University of Dusseldorf, Dusseldorf, Germany).

Patients were included following our inclusion criteria.

### 2.1. Inclusion Criteria

1. Bilateral posterior edentulism (class 1 of Kennedy classification) in one arch, or in both arches, rehabilitated with one/two removable prosthesis/es (CoCr/PMMA/Valplast-RPD) with clasps positioned on the bicuspids, that the patient had been using for at least 1 year, since more than 5 months are necessary for the patient to adapt to functional use with the new dentures, as suggested by Moreno et al. [11].
2. The prostheses were checked with polyether vinyl materials for pressure points and precision of fit and, if necessary, relined until a good fit was achieved.
3. Lack of articular/muscular, dental, or spontaneous pain.
4. Sixty-five years old, or older (according to the definition of a geriatric patient) [12].

### 2.2. Exclusion Criteria

1. The patient was suffering from psychiatric diseases or other conditions that could affect the movement.
2. The patient was suffering from periodontitis, due to possible reduced bone support and related sensory function, possible mobility and associated symptoms which could compromise chewing strength.
3. The patient had diabetes or was undergoing cancer treatment.

Three types of RPD were studied: (a) flexible Valplast-RPD (Nylon 12, Valplast International Corp, NY, USA); (b) definitive CoCr-RPD and (c) PMMA-RPD.

Therefore, the study selected 120 subjects that had only one RPD (Group 1), and 120 subjects that had two RPDs (Group 2).

In Group 1, 40 patients wearing a CoCr-RPD, 40 patients wearing a PMMA-RPD and 40 Patients wearing a V-RPD were enrolled.

In Group 2, 40 patients wearing two CoCr-RPDs, 40 patients wearing two PMMA-RPDs and 40 Patients wearing two V-RPDs were enrolled.

The MBF was measured using a digital dynamometer (KRATOS Equipamentos model IDDKv4, Sao Paulo, Brazil) with a bite fork [10]. After placing the patient in an upright position, the bite fork, covered with a new latex finger to avoid contamination, was placed on the first molar area, where 80% of the total bite force is exerted [12], and the patient was requested to bite on the device for 5 s. This measurement was repeated three times in the right arch and three times in the left arch, with two-minute intervals between each measurement.

The same operator examined all patients (relative intra-observer variability = 2%). The mean MBF was calculated in each subject and used for the statistical analysis.

Height and weight were measured to the nearest 1 cm and 0.1 kg, respectively. BMI (body mass index) was calculated using the following formula: BMI = weight (kg)/height (m)^2^.

According to the World Health Organization (WHO) criteria, patients were categorized as follows: <18.5 kg/m^2^, underweight; 18.5–24.9 kg/m^2^, normal-weight; 25.0–29.9 kg/m^2^, overweight and ≥30 kg/m^2^, obese.

### 2.3. Method Error

Method errors for numerical variables in this study were tested using the Houston formulas and the coefficient of reliability. The error ranged between 0.1% and 0.17% and the coefficient of reliability was above 92%, representing adequate agreement.

### 2.4. Statistical Analysis

Data were analyzed by ANOVA, considering gender, BMI and MBF as variables. Post hoc comparisons were performed using Fisher’s Protected Least Significant Difference (PLSD) test.

Correlation analysis of MBF values with age and BMI was performed with simple regression analysis. The level of statistical significance was set at *p* < 0.05. Statistical analysis was performed using the Statview software from SAS Institute.

## 3. Results

### 3.1. MBF and Gender

**Figure 1:**
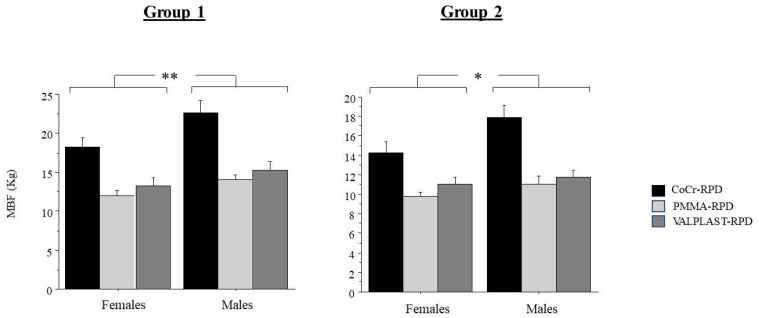
Maximal Bite Force according to gender. MBF = Maximal Bite Force; BMI = Body Mass Index; V-RPD = Valplast Removable Partial Denture; CoCr-RPD = Chrome-Cobalt Removable Partial Denture; PMMA-RPD = Polymethyl Methacrylate Removable Partial Denture. * = *p* < 0.05; ** = *p* < 0.01.

Figure 1 shows the MBF in males and females in Groups 1 and 2.

ANOVA showed a significant effect of gender for Groups 1 (*p* < 0.01) and 2 (*p* < 0.05); as males had significantly higher MBF than females.

There was no interaction of gender with the type of prosthesis for Group 1 (*p* = 0.77) and Group 2 (*p* = 0.147). The post hoc test confirmed the ANOVA test results.

### 3.2. MBF and Weight Category

**Figure 2:**
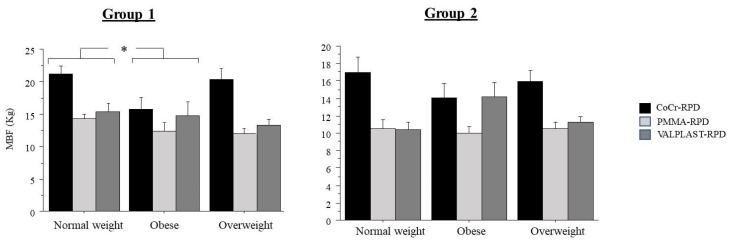
Maximal bite force according to body mass index classification. (MBF = Maximal Bite Force; BMI = Body Mass Index; V-RPD = Valplast Removable Partial Denture; CoCr-RPD = Removable Partial Denture Cobalt-Chromium; PMMA-RPD = Removable Partial Denture in Polymethyl Methacrylate.* = *p* < 0.05).

Figure 2 displays the bite force, according to the BMI categories in Groups 1 and 2. Statistical analysis showed no significant effect of weight category on MBF for Groups 1 (*p* = 0.057) and 2 (*p* = 0.969). However, post hoc test showed that in Group 1, the bite force was lower in obese subjects as compared to normal-weight subjects (*p* < 0.05). 

### 3.3. Bite Force and Prosthesis

**Figure 3:**
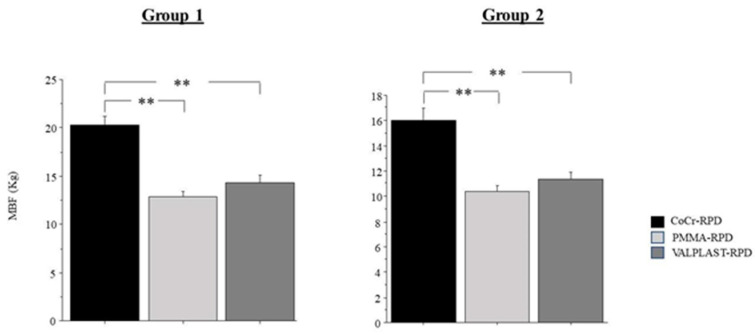
Maximal bite force in the three types of prostheses. (MBF = Maximal Bite Force; V-RPD = Valplast Removable Partial Denture; CoCr-RPD = Cobalt-Chrome Removable Partial denture; PMMA-RPD = Polymethyl Methacrylate Removable Partial Denture. ** = *p* < 0.01).

The bite force according to the type of prosthesis is shown in Figure 3. There was a significant effect of the type of prosthesis for Group 1 (*p* < 0.001) and Group 2 (*p* < 0.001). In Group 1, patients wearing CoCr-RDPs had an increased bite force as compared to those wearing PMMA-RDPs (*p* < 0.001) and Valplast-RDPs (*p* < 0.001). Similarly, in Group 2, patients wearing CoCr-RDPs had an increased bite force as compared to PMMA-RDPs (*p* < 0.001) and Valplast- RDPs (*p* < 0.001). There were no significant differences in bite force between patients wearing PMMA-RDP and Valplast-RDP in both Groups (*p* = 0.186 Group 1; *p* = 0.267 Group 2). Overall, there was a significant difference between Group 1 and 2 as Group 1 had increased bite force as compared to Group 2 (*p* < 0.001).

### 3.4. Correlation of Bite Force with Age

**Figure 4:**
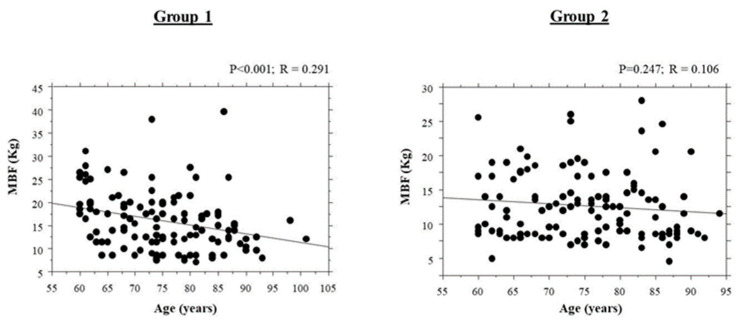
Correlations of maximal bite force with the age of the subjects. MBF = Maximal Bite Force.

The correlation of bite force with age is shown in Figure 4. There was a significant negative correlation between bite force and age for Group 1 (*p* < 0.001; coefficient of correlation (R) = 0.291), with older patients having a lower bite force. There was no correlation between bite force and age in Group 2 (*p* = 0.247; R = 0.106). In both Group 1 (*p* = 0.691) and Group 2 (*p* = 0.998), there were no significant differences in age among subjects wearing the three types of prostheses.

### 3.5. Correlations of Bite Force with Body Mass Index Values

**Figure 5:**
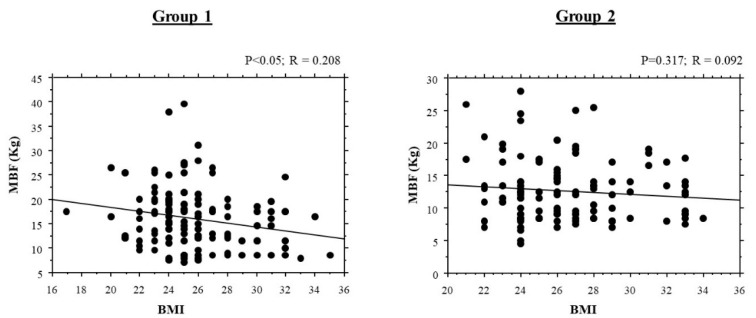
Correlations of maximal bite force with body mass index. MBF = Maximal Bite Force.

The correlation of bite force with BMI is shown in Figure 5. There was a significant negative correlation between bite force and BMI for Group 1 (*p* < 0.05; R = 0.208): the bite force decreased in subjects with higher BMI values. There was no correlation between bite force and BMI in Group 2 (*p* = 0.317; R = 0.092).

In the present sample, only 63.7% of males and 35% of females wearing one CoCr-RPD, 20% of males and 10% of females wearing one V-RPD and 35% of males and 5% of females wearing two CoCr-RPDs had a UBF higher than 8 kg: therefore, patients wearing one, or two, CoCr-RPDs had a much higher chance of expressing the bite force needed to keep a healthy eating plan (Table 1).

Although this evaluation is only indicative, it can be useful for understanding the difficulties that people meet in eating when wearing some kinds of RPD, such as PMMA-RPD and V-RPD.

Our study shows a significant negative correlation between bite force and BMI for Group 1 (*p* < 0.05; R = 0.208) where the bite force decreased in subjects with higher BMI values. These data confirm that a better chewing function leads to the intake of healthier foods. Soft food is easier to chew but can cause an increase in BMI and the consequent risks of metabolic syndrome, obesity, diabetes, and cardiovascular diseases, while at the same time not providing basic nutrients, such as proteins, fibers and vitamins.

In the current study, people wearing CoCr-RPDs had the highest MBF, while at the same time, showed the lowest BMI and percentage of overweight subjects than other Groups (Table 2).

## 4. Discussion

Masticatory efficiency is key to avoiding a reduction in food intake and ensuring appropriate nutrition in the elderly. The key elements of efficient chewing are the number of healthy teeth, the number of premolars/molars occlusal contacts and the bite force. People with more than 20 teeth have a high bite force and sufficient masticatory abilities [13]. In extremely shortened arches, with less than three occluding premolars, chewing ability is severely impaired [14].

People with insufficient masticatory strength compensate by chewing for a longer time and by eating texture-modified food, such as pureed food, which is easier to chew and swallow, or by swallowing coarser particles [4].

In the present study, the relationship between MBF and BMI and different RPD was investigated. Measurement of voluntary MBF is a simple and quick way to assess the functional state of the masticatory system [8] and to evaluate masticatory ability. The present study found that MBF tends to decrease significantly with age, similarly to what Takaki et al. [15] have found. Older patients have a lower bite force, and this is mainly caused by the muscular atrophy that physiologically happens with aging, but it might be correlated to a change in diet, eating softer foods, and because of the development of partial, or total, edentulism, therefore diminishing the training of the jaw muscles and causing atrophy.

Males showed higher MBF values than females as found by other authors [13]. These differences are possibly due to the larger muscular mass in males than in females, as highlighted by computer tomography [16]. The highest MBF average value was shown by patients wearing CoCr-RPDs, followed by PPMA-RPDs and V-RPDs. The difference is not significant between the last two. This is due to the alloy’s higher mechanical properties that achieve better stability and retention than other materials. Their clasps and rests contact the abutment teeth and are rigid enough to distribute masticatory forces over the entire dental arch [17], ensuring optimal stability and a strong occlusal force.

PMMA-RPDs have a lower elastic modulus than CoCr-RPDs and flex under mastication [17]; V-RPDs have the lowest elastic modulus, and their clasps offer less retention due to their distortion during use; also, their high deformability bring a larger load onto the mucosa under the denture [3].

Because of the lack of rests on the teeth surfaces, PMMA-RPDs and V-RPDs tend to sink under mastication and can cause erythema and pain mainly around the abutment teeth. These problems are emphasized during the MBF test and restrict the closure force. No pain was claimed by CoCr-RPDs wearers during the test.

Subjects wearing V-RPDs showed higher MBF value than PMMA-RPDs despite the lower retentive force of the clasps, in contrast to what Macura-Karbownik et al. [4] found.

This difference, although not significant, may be related to the higher adhesive capacity of Valplast during chewing [18], and to a hypothesized better stability of thermoplastic materials compared to conventional PMMA, due to its elasticity.

In Group 2, MBF decreases significantly compared to Group 1. These data indicate a worse masticatory situation when both arches are wearing RPDs. In this Group CoCr-RPDs show again the best results. 

Studies showed that different amounts of bite force are needed for different kinds of food. For example, to appropriately penetrate rye bread, 16.7 kg are needed, while in order to penetrate raw cabbage 7.4 kg are needed: 8 kg is usually defined as the minimum value needed to guarantee a satisfactory biting force, in order to maintain a complete diet that includes meat and most vegetables and fruits [19,20]. 

Every subject has his/her own masticatory force related to gender and genetic factors, but the highest MBF value that one can express is not reached during habitual chewing, which occurs at 40% of their MBF [21].

As mentioned in the results, in the present sample, only 63.7% of males and 35% of females wearing one CoCr-RPD, 20% of males and 10% of females wearing one V-RPD and 35% of males and 5% of females wearing two CoCr-RPDs had a UBF higher than 8 kg: therefore, patients wearing one, or two, CoCr-RPDs had a much higher chance of expressing the bite force needed to keep a healthy eating plan. This can be useful for understanding the difficulties that people meet in eating when wearing some kinds of RPD, such as PMMA-RPD and V-RPD.

Our study shows a significant negative correlation between bite force and BMI for Group 1 (*p* < 0.05; R = 0.208) where the bite force decreased in subjects with higher BMI values. These data confirm that a better chewing function leads to the intake of healthier foods. Soft food is easier to chew but can cause an increase in BMI and in the consequent risks of metabolic syndrome, obesity, diabetes, and cardiovascular diseases, while at the same time not providing basic nutrients such as proteins, fibers and vitamins.

In the current study, people wearing CoCr-RPDs had the highest MBF, while at the same time, showed the lowest BMI and percentage of overweight subjects than other Groups. RPD treatment increases the number of occluding posterior teeth, improving the chewing efficiency, but the results are not the same for all kinds of RPDs.

Only CoCr-RPDs can guarantee a satisfactory bite force, as PMMA-RPDs and V-RPDs failed to functionally rehabilitate a Kennedy 1st class. The worst MBF values were observed when RPDs were utilized in both arches.

Although not statistically significant, subjects wearing a CoCr-RPD showed a lower mean weight, a lower BMI and a slightly lower percentage of overweight subjects compared to those who wear a PMMA-RPD or a V-RPD. This might imply diversity in diet and choice of food. While RPDs can protect partially edentulous patients against the risk of malnutrition, our findings suggest that only CoCr-RPDs may ensure satisfying nutrition.

PMMA-RPDs and V-RPDs should therefore not be considered as an appropriate definitive rehabilitation, and, when possible, an implant-supported fixed prosthesis should be adopted to restore the proper masticatory function [22,23,24,25].

V-RPDs are clearly more esthetically satisfactory than other kinds of RPDs, thanks to tooth-colored retentive clasps and rests, and their demand is clearly growing. Given our findings, we advise only using them in replacing anterior teeth, or posterior teeth in subjects with very low masticatory forces.

This study has some limitations in design and method. Other parameters, such as cross-bite and degree of initial resorption of the edentulous ridges could be included and correlated in the evaluation. In addition, it was not possible to randomize the sample because clinical, aesthetic and economic criteria for the choice of the prosthesis were followed on a patient-by-patient basis.

In the present study, only the MBF was evaluated as an index of masticatory efficiency; the MBF can only describe the vertical component of the masticatory cycle. Bite forces occur not only in the vertical direction but also in the transverse directions. Therefore, further three-dimensional force measurements are necessary to obtain a complete analysis of masticatory patterns in RPDs wearers.

## 5. Conclusions

An appropriate masticatory function is needed to maintain a correct nutritional plan, therefore avoiding several related diseases, preserving neurocognitive functions in the elderly, and therefore limiting public expenditure.

Considering that habitual mastication is 40% of MBF, the present results show that only CoCr-RPD wearers are able to chew more protein-rich food, whereas PMMA-RPD and V-RPD, due to the materials they are made of, their instability and the associated pain caused during mastication, usually limit patients to only eat more soft, easier to chew, foods.

The lowest MBF was found in subjects wearing V-RPDs and PMMA-RPDs in both arches. In these cases, patients suffer from a severe masticatory deficiency, and can only chew softer foods, which provide very few nutrients, while at the same time causing an increase in BMI and therefore in metabolic diseases.

It can be concluded that there is a correlation between bite forces, which are directly related to chewing efficiency, and the type of removable denture. Despite all RPDs re-establishing posterior occlusal contacts, only CoCr-RPDs restore an objectively satisfying chewing function and therefore can influence patients’ diet. Otherwise, implant-supported rehabilitation should be adopted to properly restore the masticatory function.

## Figures and Tables

**Table 1:** Maximum Bite Forces in the 6 subgroups. [MBF = Maximum Bite Force (kg); UBF = Usual bite force (40% of MBF, threshold value 8 kg), CoCr-RPD = cobalt-chrome removable partial denture; PMMA-RPD = polymethyl methacrylate removable partial denture; Valplast-RPD = Valplast removable partial denture].

	Type of Prosthesis	Gender	MBF Higher Value	MBF Lower Value	MBF Mean Value	*N* of Subjects (%) with a UBF > 8 kg
GROUP 1	CoCr-RPD	males	39.5	12	22	14 (63.7%)
		females	26.5	8.5	17.5	9 (35%)
	PMMA-RPD	males	1.5	8.5	14	0
		females	17	7	11	0
	V-RPD	male	24.5	7.5	15	4 (20%)
		females	21.5	8.5	13	2 (10%)
GROUP 2	CoCr-RPD	males	28	8.5	18	7 (35%)
		females	25.5	4.5	14	1 (5%)
	PMMA-RPD	males	17.5	5	11	0
		females	13.5	7.5	10	0
	V-RPD	males	19	7	11	0
		females	1.7	7	11	0

**Table 2:** Body Mass Index in the 6 subgroups. [MBF = Maximum Bite Force (kg); UBF = Usual bite force (40% of MBF, threshold value 8 kg), CoCr-RPD = cobalt- chrome removable partial denture; PMMA-RPD = polymethyl methacrylate removable partial denture; Valplast-RPD = Valplast removable partial denture].

	Type of Prosthesis		Gender	Max Value	Min Value	Mean Value	*N* of Overweight Subjects (%)	*N* of Obese Subjects
GROUP 1 RPD/TEETH		CoCr-RPD	males	32	21	25.1	12 (54%)	2
			females	34	20	25	14 (53%)	3
		PMMA- RPD	males	33	17	26.2	13 (65%)	5
			females	33	21	25.9	14 (63%)	3
		Valplast- RPD	male	31	23	26.3	13 (65%)	4
			females	30	21	25.7	12 (60%)	3
GROUP 2 RPD/RPD		CoCr-RPD/ CoCr-RPD	males	33	21	26.2	13 (65%)	4
			females	30	22	25.9	12 (60%)	2
		PMMA-RPD/PMMA- RPD	males	33	24	27.2	14 (70%)	4
			females	31	22	26.8	13 (65%)	4
		V-RPD/ V-RPD	males	33	23	26.8	13 (70%)	3
			females	34	22	26.1	13 (65%)	3

## Data Availability

Not applicable.
